# Necrotizing Fasciitis by Two Anaerobic Bacteria in an Immunocompetent Patient after Minor Trauma: A Case Report

**DOI:** 10.1155/2018/4910292

**Published:** 2018-09-09

**Authors:** Marco Sciarra, Andrea Schimmenti, Tommaso Manciulli, Cristina Sarda, Marco Mussa, Laura Sacco, Bianca Mariani, Angela Maria Di Matteo, Paolo Orsolini

**Affiliations:** ^1^Department of Internal Medicine, University of Pavia, Pavia, Italy; ^2^PhD in Experimental Medicine, University of Pavia, Pavia, Italy; ^3^Department of Clinical, Surgical, Diagnostic and Pediatric Sciences, University of Pavia, Pavia, Italy; ^4^Unit of Microbiology and Virology, IRCCS San Matteo Hospital Foundation, Pavia, Italy; ^5^Unit of Infectious and Tropical Diseases, IRCCS San Matteo Hospital Foundation, Pavia, Italy

## Abstract

Necrotizing fasciitis (NF) is a soft tissue infection affecting subcutaneous tissue and the muscular fascia without involvement of the muscle and can be either monomicrobial or polymicrobial. Monomicrobial infections are usually caused by group A streptococci, while infections caused by anaerobic germs usually affect immunodepressed patients. We report a rare case of NF caused by two anaerobic bacteria in an immunocompetent patient.

## 1. Case Presentation

**Figure 1:**
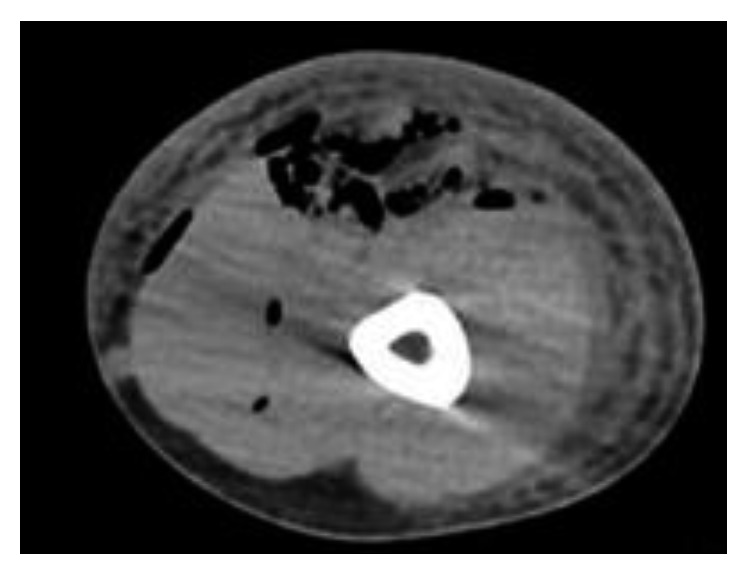
CT scan of the patient's left arm performed at the emergency department showing the presence of subcutaneous emphysema.

A 40-year-old Caucasian man was admitted to our ward. He reported being a 6 pack/year smoker, occasional drinker, and had an otherwise unremarkable medical history. He presented with two accidentally self-inflicted wounds on the left arm caused by the mishandling of two work tools a week prior. One episode occurred while disposing of a metallic tool used during his morning work as a plumber and the second one in the afternoon with a metal needle used in the breeding of carps that he practices as a hobby. The day before admission, the patient presented at the Emergency Department (ED) of a hospital near his residence with redness and swelling in the left upper arm and fever. He was prescribed a seven-day course of amoxicillin/clavulanate 875 + 125 mg PO TID and discharged. He came back to the same ED the next day for excruciating pain in the entire arm, persistent fever, and extension of edema. A soft tissue ultrasound scan showed lymphedema of the left arm without signs of thrombosis. He was then referred to the Emergency Department of our hospital for further diagnostic investigations on 15/06/2017 which included an Infectious Disease consult who suspected necrotizing fasciitis (NF). A contrast-enhanced CT showed thrombosis and subcutaneous emphysema (Figure 1). The patient was then started on a broad-spectrum empirical antibiotic therapy with metronidazole 500 mg IV QID, piperacillin/tazobactam 4.5 g IV TID, and doxycycline 100 mg PO BID. An orthopedic surgeon was consulted, and fasciotomy followed by surgical debridement was immediately performed. Blood cultures were obtained before the start of antibiotic therapy using two sets of bottles for aerobes and anaerobes, incubated in the automated system Bacted^TM^ FX (BD, Franklin Lakes, NJ, USA). Surgical samples from muscular bands and cutaneous flaps obtained during the fasciotomy were collected using an eSwab^TM^ (COPAN Diagnostics, Murrieta, CA, USA) kit and cultured on different types of agar (blood, chocolate, McConkey, Mannitol, and Schädler). A GENbag anaer® (bioMérieux, Marcy l'Etoile, France) kit was also used to detect anaerobic bacteria. Antimicrobial susceptibility testing was carried out on Mueller–Hinton medium supplemented with 5% Fildes blood digest. The patient was then transferred to the Infectious Disease Department, and antibiotic therapy was restricted to piperacillin/tazobactam plus vancomycin, in accordance with the current IDSA guidelines [1]. A vacuum-assisted closure (VAC) therapy cycle was also started on 23/06/2017.

**Figure 2:**
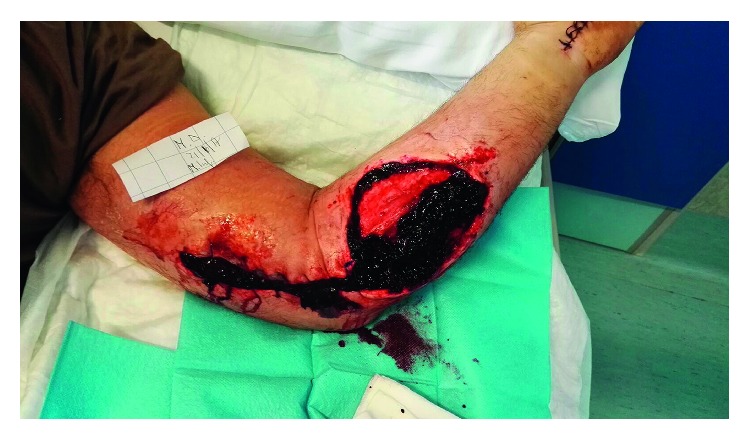
Patient's left arm after an initial round of antibiotic treatment.

**Figure 3:**
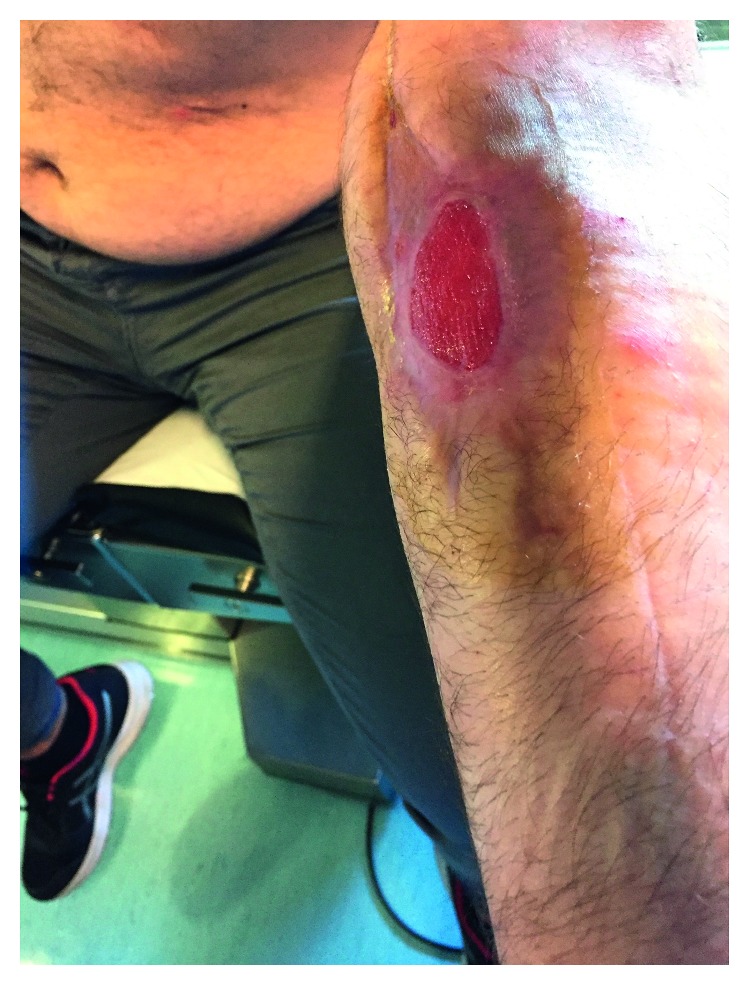
Patient's arm during an outpatient checkup performed after the patient underwent plastic surgery for skin grafting, showing the almost complete resolution of FN.

**Figure 4:**
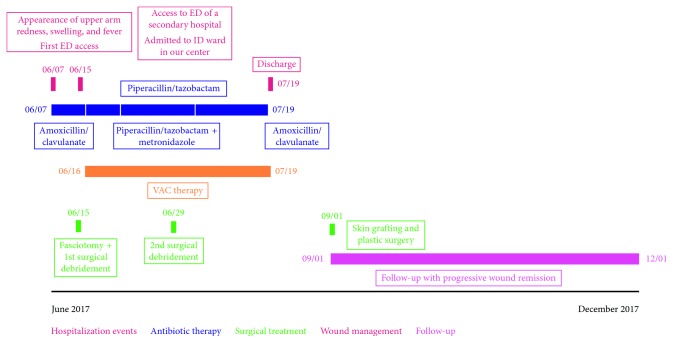
Timeline summarizing the presented case.

The cultures of eSwab samples and surgical samples became positive on 25/06/2017 and 26/06/2017. *Peptostreptococcus anaerobius* and *Atopobium parvulum,* two anaerobic pansensitive bacteria, were isolated and identified by matrix-assisted laser desorption ionization-time of flight (MALDI-TOF), using the Bruker database as reference for the microorganism identification process (Bruker Daltonics GmbH, Bremen, Germany). The isolates returned susceptible to piperacillin/tazobactam, and the patient continued his treatment (Table 1). Despite the use of appropriate antibiotic therapy and the use of advanced medications and VAC therapy, a large area of coagulation and necrosis remained in the affected area (Figure 2). A new debridement was performed on 29/06/2017, and the patient continued the VAC therapy. Subsequently, granulation tissue began to form. Antibiotic therapy was changed to oral amoxicillin/clavulanate 1 g TID PO on 07/05/2017. The patient was discharged on 19/07/2017. He then underwent plastic surgery for autologous skin grafting, and after three months, the lesion had almost disappeared (Figure 3).  Figure 4 summarizes the events detailed in this case presentation.

## 2. Discussion

Necrotizing fasciitis is a soft tissue infection affecting the muscular fascia and subcutaneous tissues without involvement of the muscle [1, 2]. Microbiologically, there is a distinction between type 1 fasciitis, also called polymicrobial as it is caused by two different germs, and type 2 or monomicrobial fasciitis [1, 3]. Usually, the latter is due to group A *Streptococcus* [4, 5]. This infection is one of the few urgent conditions in the field of Infectious Diseases [1–3]. This is due to its rapid clinical course and high mortality rates: as many as 21% of patients with type 1 and 14 to 39% with type 2 fasciitis die [3]. Trauma is a known risk factor for this infection. Interestingly, reports of NF have related to both minor and major traumas (insect bites, scratches, abrasions, minor burns, lacerations, and surgery) [2, 6]. Conditions such as immunosuppression, diabetes, chronic renal failure, peripheral vascular disease, cirrhosis, neutropenia, obesity, and intravenous drug abuse are associated with an increased risk of developing NF [2, 6].

In this patient, two anaerobic pansensitive microorganisms, *Peptostreptococcus anaerobius* and *Atopobium parvulum*, were isolated. These anaerobic bacteria are usually part of the normal oral and gut flora of humans and animals [7, 8]. The former is frequently associated with diabetic surgical wound infection and necrotizing fasciitis [9, 10], while *A. parvulum* is rarely associated with necrotizing fasciitis [11]. Our patient presented with anaerobic NF despite being young, nondiabetic, and not having risk factors for NF except for the presence of minor wounds. To our knowledge, this is the first report of these two scarcely virulent bacteria as etiological agents of anaerobic necrotizing fasciitis in a young immunocompetent man.

## Figures and Tables

**Table 1:** Resistance profile of the two isolated bacteria.

*P. anaerobius*	
Antibiotic	RSI
Penicillin	S
Ampicillin	S
Amoxicillin/clavulanate	S
Piperacillin/tazobactam	S
Metronidazole	S
Imipenem	S
Meropenem	S
Clindamicyn	S
Vancomycin	S

*A. parvulum*	
Penicillin	S
Ampicillin	S
Amoxicillin/clavulanate	S
Piperacillin	S
Piperacillin/tazobactam	S
Metronidazole	S
Imipenem	S
Meropenem	S
Clindamicyn	S
Vancomycin	S
